# Supplementary material for: Recombinant Peptide Production Softens Escherichia coli Cells and Increases Their Size during C-Limited Fed-Batch Cultivation
Source: Int J Mol Sci. 2023 Jan 30;24(3):2641. doi: 10.3390/ijms24032641 (PMC9916741; doi:10.3390/ijms24032641)
Supplement: Supplementary file 1 [file ijms-24-02641-s001.zip › ijms-1867875-supplementary.pdf]

Recombinant peptide production softens *Escherichia coli* cells and increases their size during C-limited fed-batch cultivation

Supplementary materials

Authors: Andreas Weber[†,1,2], Martin Gibisch[†,1], Daniel Tyrakowski[2], Monika Cserjan-Puschmann[1], José L. Toca-Herrera[2,*] Gerald Striedner[1]

Affiliations:

1 Christian Doppler Laboratory for production of next-level biopharmaceuticals in *E. coli*, Institute of Bioprocess Science and Engineering, Department of Biotechnology, University of Natural Resources and Life Sciences, Vienna, Muthgasse 18, 1190 Vienna, Austria

2 Institute of Biophysics, Department of Nanobiotechnology, University of Natural Resources and Life Sciences; Muthgasse 11, 1190 Vienna, Austria.

† These authors have contributed equally.

* Corresponding author.

Keywords: Peptide expression, Bioprocess engineering, Bacteria, Periplasmic space, Viscoelasticity, Biophysics, Membrane properties, Atomic Force Microscopy

A

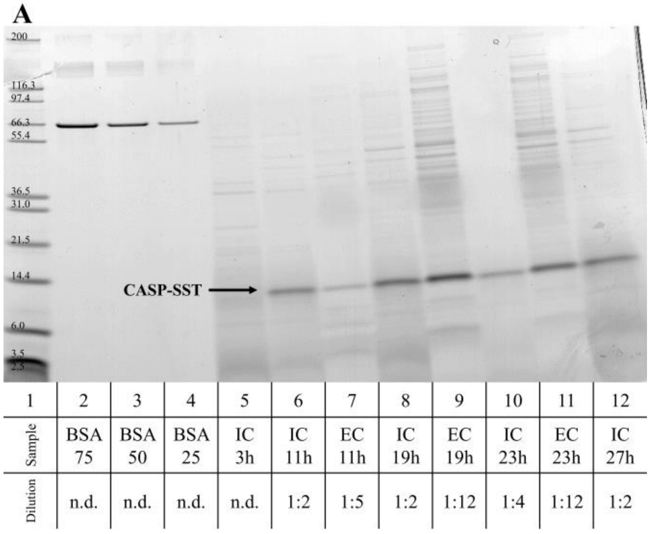

1	2	3	4	5	6	7	8	9	10	11	12
Sample	BSA 75	BSA 50	BSA 25	IC 3h	IC 11h	EC 11h	IC 19h	EC 19h	IC 23h	EC 23h	IC 27h
Dilution	n.d.	n.d.	n.d.	n.d.	1:2	1:5	1:2	1:12	1:4	1:12	1:2

B

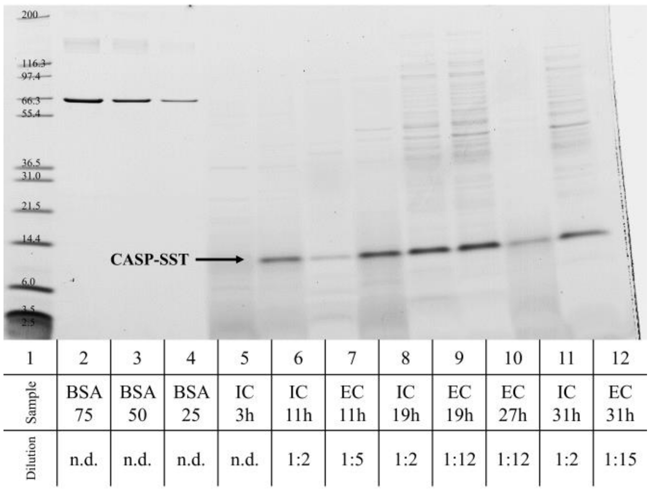

1	2	3	4	5	6	7	8	9	10	11	12
Sample	BSA 75	BSA 50	BSA 25	IC 3h	IC 11h	EC 11h	IC 19h	EC 19h	EC 27h	IC 31h	EC 31h
Dilution	n.d.	n.d.	n.d.	n.d.	1:2	1:5	1:2	1:12	1:12	1:2	1:15

C

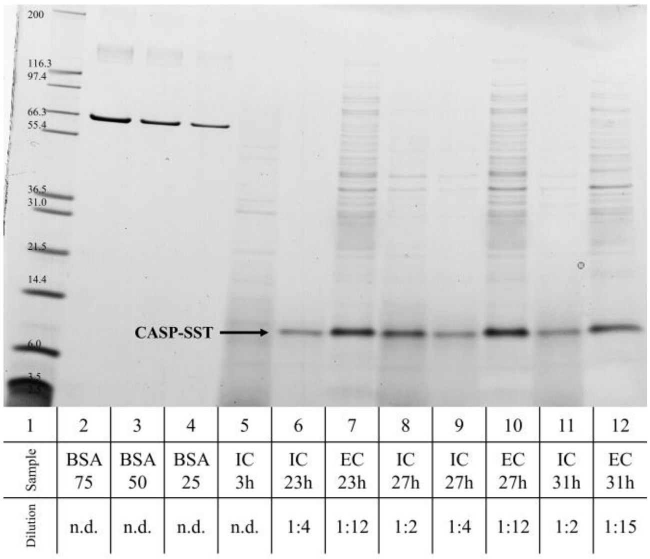

1	2	3	4	5	6	7	8	9	10	11	12
Sample	BSA 75	BSA 50	BSA 25	IC 3h	IC 23h	EC 23h	IC 27h	IC 27h	EC 27h	IC 31h	EC 31h
Dilution	n.d.	n.d.	n.d.	n.d.	1:4	1:12	1:2	1:4	1:12	1:2	1:15

Figure S1. SDS-PAGE gels (panels **A–C**) for quantification of intracellular (IC) and extracellular (EC) peptide content. All samples were analysed at least twice. 3 h samples represent uninduced reference samples. CASP-SST bands (black arrow) were quantified via ImageQuant™ using BSA standards (lanes

2-4, 75-25 µg mL^{-1}). Samples were diluted to fit the linear range of the standard, and non-diluted samples are indicated as n.d.

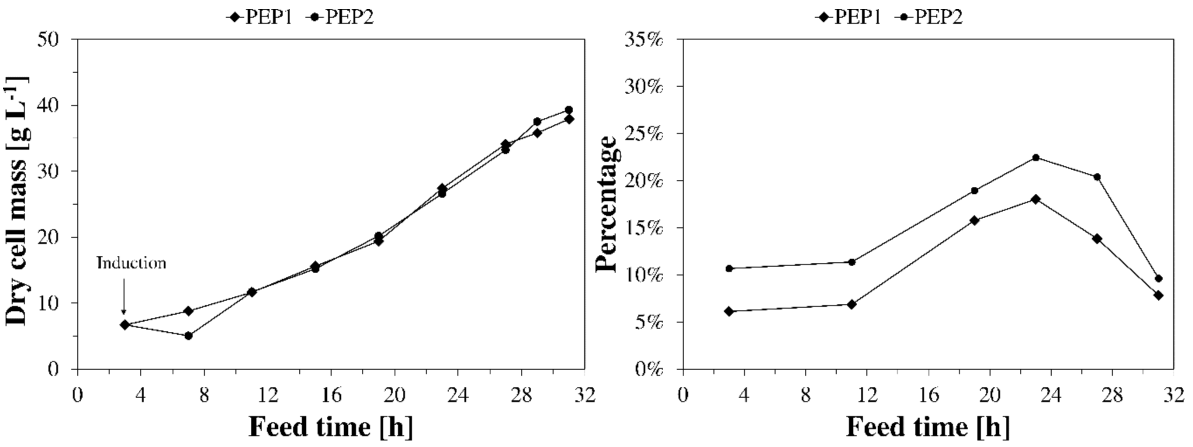

Figure S2: Growth curves (**A**) and cell lysis curves (**B**) of duplicate cultivations with PEP. Minor deviations in DCM (e.g. after 7 h) likely arise from errors during sample preparation. Cell lysis variation might arise from differences in timepoint of sample preparation and DNA measurement (DNA instability).

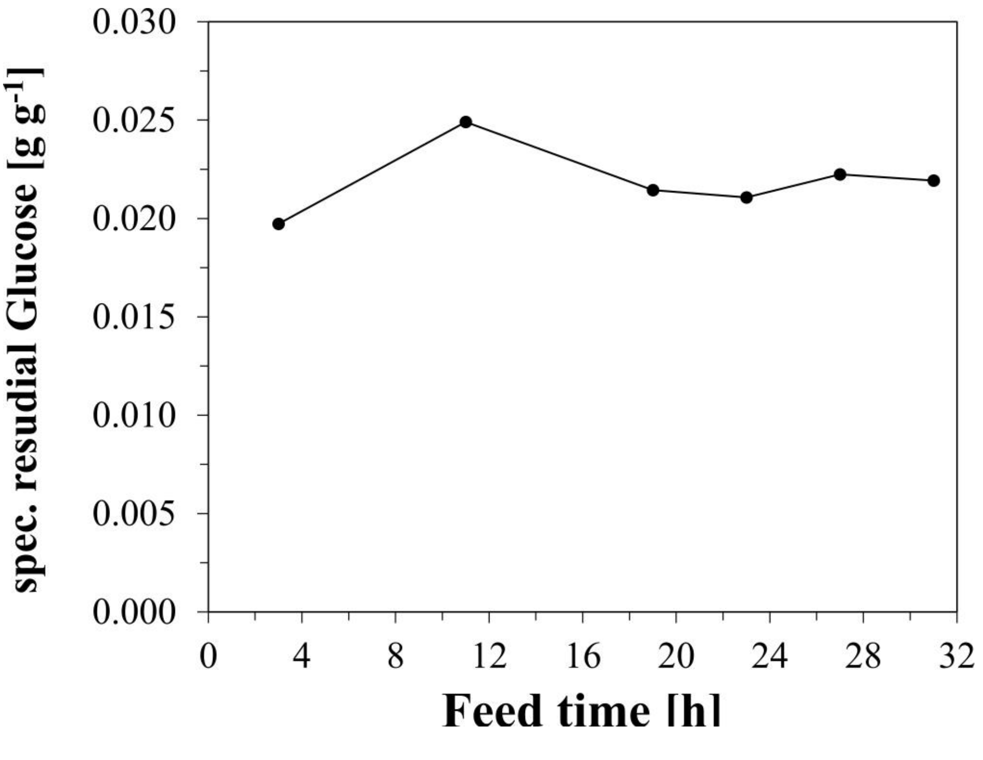

Figure S3. Specific residual glucose concentration in the cell-free cultivation medium of PEP. Glucose concentrations were analysed using HPLC as previously described by Marisch et al., 2013 (https://doi.org/10.1186/1475-2859-12-58).

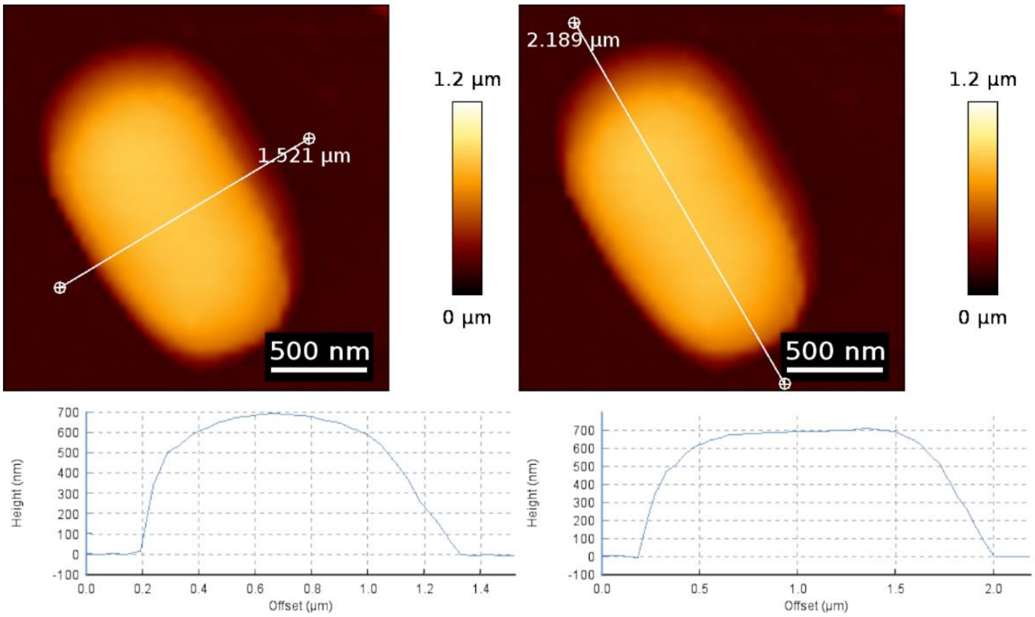

Figure S4. Analysis of cell width (**left**) and cell length (**right**) from AFM images.

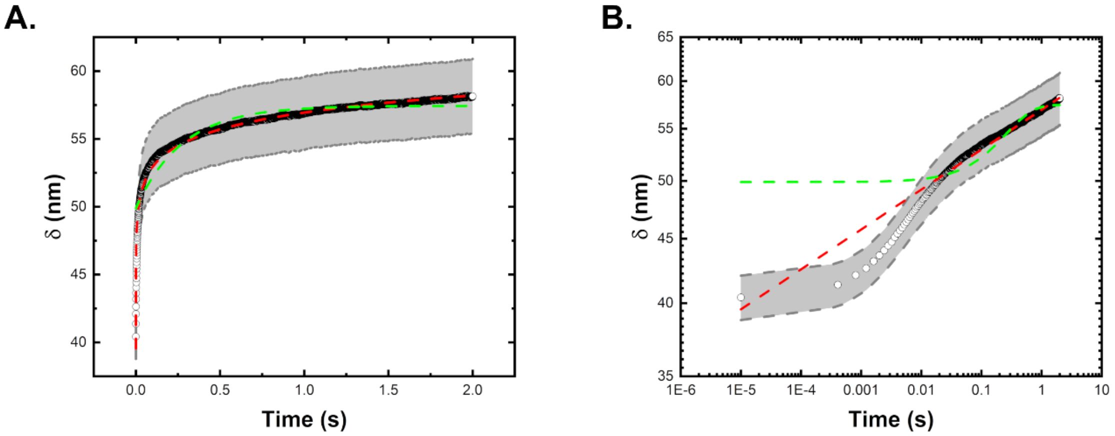

Figure S5. Performance of fitting procedures for creep curves (red power law, green standard linear solid) in normal representation (**A**) and logarithmic representation (**B**).

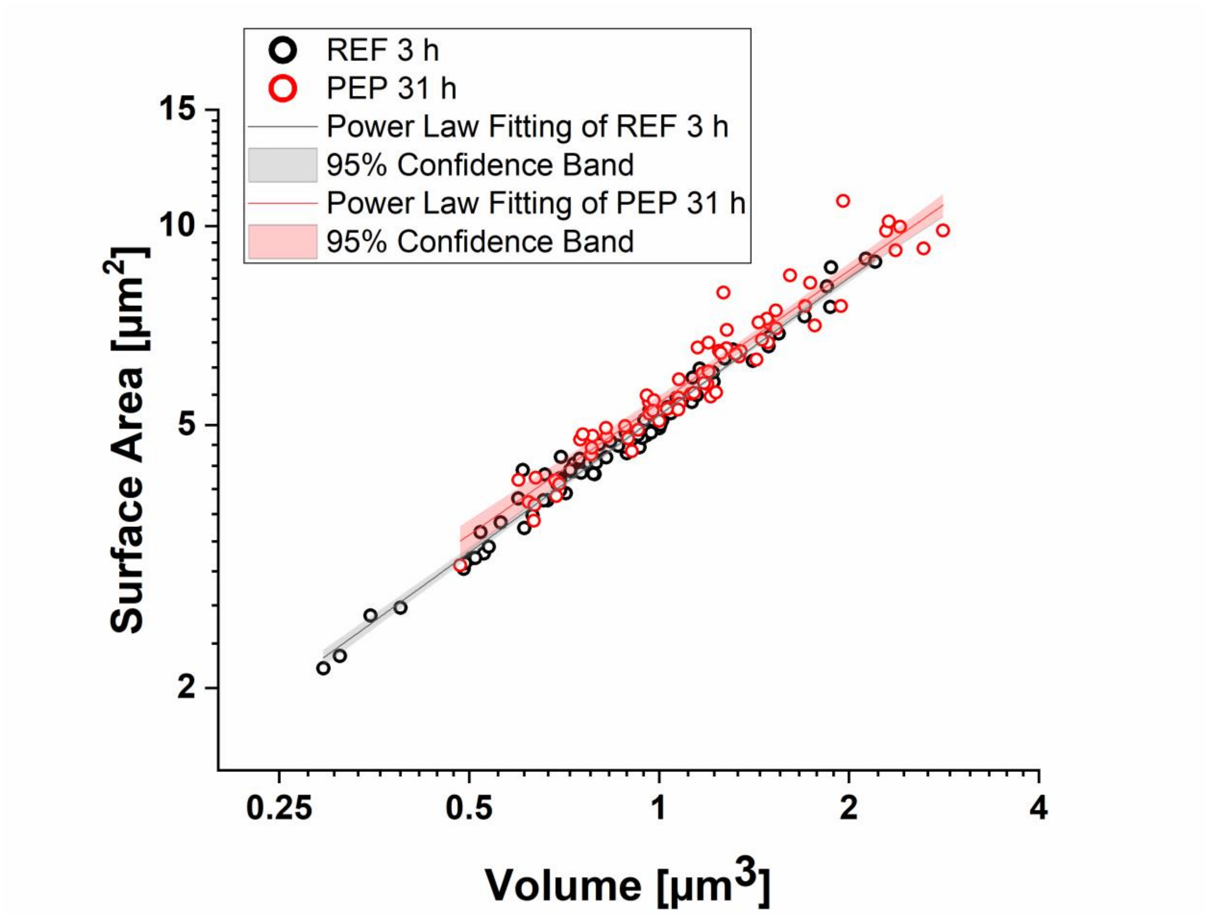

Figure S6. Surface area to volume scaling is conserved for the different samples. Here shown is REF 3h and PEP 31 h.

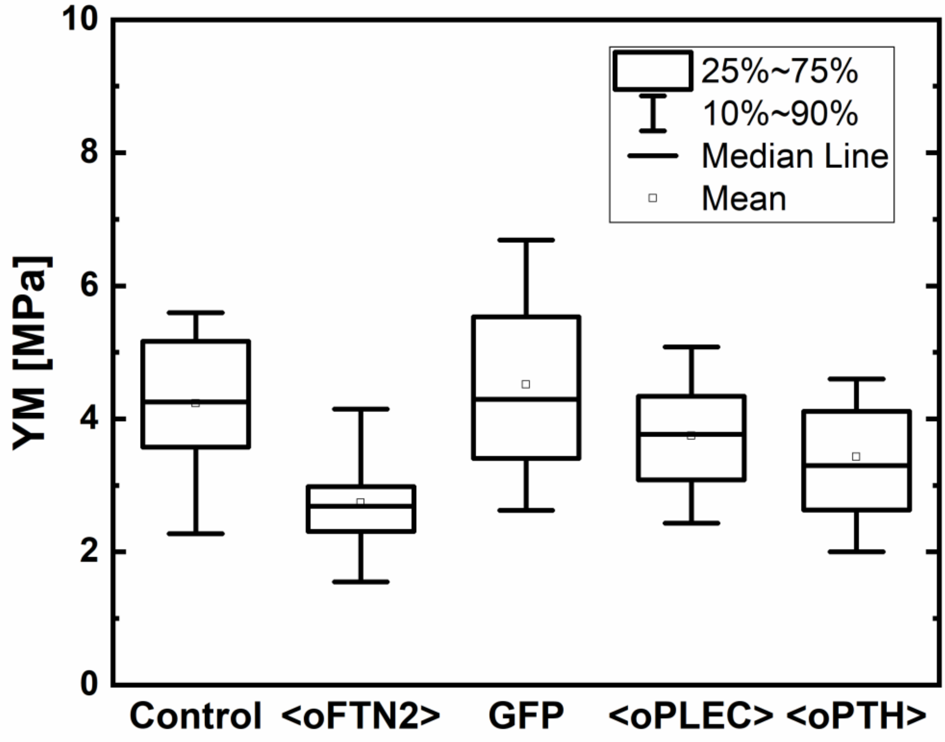

Figure S7. Apparent Young's Modulus of BL21(DE3) cells expressing recombinant Fab fragments (FTN2), green fluorescent protein (GFP), antimicrobial peptide plectasin (PLEC), and parathyroid hormone (PTH). Measurements of BL21(DE3) cells without any protein/peptide production are indicated as control. Brackets (< >) indicate genomic integration of the recombinant gene into the host. Translocation into the periplasmic space via the OmpA signal sequence of the respective protein/peptide is indicated as "o". Respective cultivations were carried out as described in the material and method section.
